# (*S*)-1-Hydroxy­propan-2-aminium (2*R*,3*R*)-3-carb­oxy-2,3-dihydroxy­propanoate monohydrate

**DOI:** 10.1107/S1600536808000391

**Published:** 2008-01-11

**Authors:** Xin-Yu Tang, Xi-Long Yan, Ping Zhang, Ling Qin, Yueguang Yin

**Affiliations:** a School of Chemical Engineering and Technology, Tianjin University, Tianjin 300072, People’s Republic of China; b Tianjin Foreign Studies University, Tianjin 300204, People’s Republic of China; c Tianjin EPC Petrochemical Engineering Co. Ltd, Tianjin 300000, People’s Republic of China

## Abstract

The chiral title compound, C_4_H_10_NO^+^·C_4_H_5_O_6_^−^·H_2_O, is a hydrated mol­ecular salt in which the tartaric acid has transferred one proton to the (*S*)-2-amino­propan-1-ol mol­ecule. The crystal structure is stabilized by a three-dimensional network of N—H⋯O and O—H⋯O hydrogen bonds. The absolute configuration was assigned on the basis of the starting materials.

## Related literature

For the synthesis, see: Bai *et al.* (2004 ▶); For background, see: Humljan *et al.* (2006 ▶).
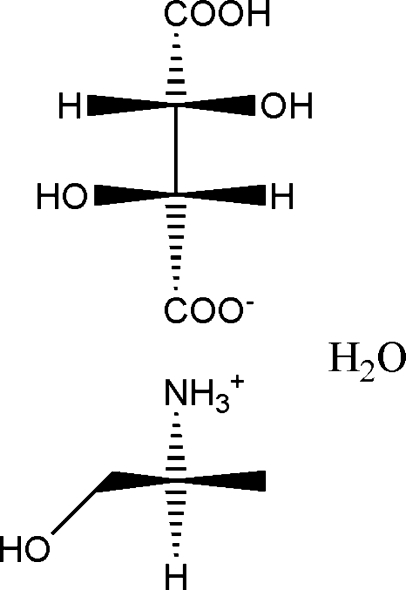


## Experimental

### 

#### Crystal data

C_4_H_10_NO^+^·C_4_H_5_O_6_^−^·H_2_O
*M* *_r_* = 243.22
Orthorhombic, 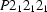

*a* = 7.533 (2) Å
*b* = 7.701 (2) Å
*c* = 19.288 (5) Å
*V* = 1118.9 (5) Å^3^
*Z* = 4
Mo *K*α radiation
μ = 0.13 mm^−1^
*T* = 294 (2) K
0.24 × 0.22 × 0.18 mm

#### Data collection

Bruker SMART CCD diffractometer
Absorption correction: multi-scan (*SADABS*; Bruker, 1997 ▶) *T*_min_ = 0.969, *T*_max_ = 0.977
6331 measured reflections
1359 independent reflections
1280 reflections with *I* > 2σ(*I*)
*R*_int_ = 0.024

#### Refinement

*R*[*F* ^2^ > 2σ(*F* ^2^)] = 0.028
*wR*(*F* ^2^) = 0.074
*S* = 1.06
1359 reflections
174 parameters
H atoms treated by a mixture of independent and constrained refinement
Δρ_max_ = 0.25 e Å^−3^
Δρ_min_ = −0.17 e Å^−3^

### 

Data collection: *SMART* (Bruker, 1997 ▶); cell refinement: *SAINT* (Bruker, 1997 ▶); data reduction: *SAINT*; program(s) used to solve structure: *SHELXS97* (Sheldrick, 2008 ▶); program(s) used to refine structure: *SHELXL97* (Sheldrick, 2008 ▶); molecular graphics: *SHELXTL* (Bruker, 1997 ▶); software used to prepare material for publication: *SHELXTL*.

## Supplementary Material

Crystal structure: contains datablocks I, global. DOI: 10.1107/S1600536808000391/hb2687sup1.cif

Structure factors: contains datablocks I. DOI: 10.1107/S1600536808000391/hb2687Isup2.hkl

Additional supplementary materials:  crystallographic information; 3D view; checkCIF report

## Figures and Tables

**Table 1:** Hydrogen-bond geometry (Å, °)

*D*—H⋯*A*	*D*—H	H⋯*A*	*D*⋯*A*	*D*—H⋯*A*
O1—H1⋯O8^i^	0.89 (3)	1.78 (3)	2.677 (2)	176 (3)
O4—H4⋯O3^ii^	0.89 (2)	2.01 (3)	2.885 (2)	169 (2)
O5—H5⋯O2^iii^	0.82 (3)	1.87 (3)	2.676 (2)	167 (3)
O6—H6⋯O2^iv^	0.85 (3)	1.77 (3)	2.6091 (19)	173 (3)
N1—H1*D*⋯O1^v^	0.93 (3)	2.05 (3)	2.945 (2)	159 (2)
N1—H1*E*⋯O5^iii^	0.95 (3)	1.91 (3)	2.852 (2)	168 (2)
N1—H1*F*⋯O6^i^	0.95 (3)	2.26 (3)	3.121 (2)	150 (2)
O8—H8*A*⋯O3	0.85 (3)	1.95 (3)	2.784 (2)	169 (3)
O8—H8*B*⋯O4^vi^	0.81 (3)	2.06 (3)	2.865 (2)	173 (3)

Symmetry codes: (i) 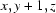; (ii) 
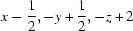
; (iii) 
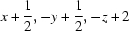
; (iv) 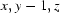; (v) 
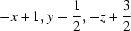
; (vi) 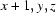.

**Fig. 1.**
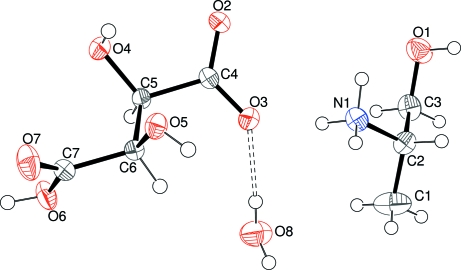
A view of the molecular structure of (I). Displacement ellopsoids are drawn at the 50% probability level and H atoms are shown as spheres of arbitrary radius. The hydrogen bond is indicated by a double dashed line.

## supplementary crystallographic information

### Comment

The title compound, (I), (Fig. 1), is a hydrated (2*R*,3*R*)-tartrate salt of (*S*)-2-aminopropan-1-ol. (*S*)-2-aminopropan-1-ol is a key intermediate for the synthesis of potential inhibitors of the bacterial peptidoglycan biosynthesis enzymes MurD and MurE (Humljan *et al.*, 2006).

In the crystal, the (*S*)-2-aminopropan-1-ol molecule is in a cationic form, and has a positively charged amino group. The tartaric acid molecule is a semi-tartrate ion, with a neutral carboxylic acid group at one end and a negatively charged carboxylate group at the other (Fig. 1). The bond distances and angles in the cation and the anion are normal. The chiralities of the carbon atoms (C2 S, C5 *R*, C6 *R*) were assigned according to the known absolute structures of the starting materials.

In the crystal structure of (I), an extensive hydrogen-bond network is built up (Table 1).

### Experimental

The title compound was prepared by the procedure of Bai *et al.* (2004). Colourless single crystals of (I) were grown by slow evaporation of a solution of methanol and water.

### Refinement

Anomalous dispersion was negligible and Friedel pairs were merged before refinement.

The N– and O-bound H atoms were located in difference maps and their positions were freely refined with *U*_iso_(H) = 1.5*U*_eq_(carrier).

The C-bound H atoms were positioned geometrically (C—H = 0.96–0.98 Å) and refined as riding with *U*_iso_(H) = 1.2*U*_eq_(C).

### Crystal data

**Table:**

C_3_H_10_NO^1+^·C_4_H_5_O_6_^1–^·H_2_O	*F*_000_ = 520
*M**_r_* = 243.22	*D*_x_ = 1.444 Mg m^−^^3^
Orthorhombic, *P*2_1_2_1_2_1_	Mo *K*α radiation λ = 0.71073 Å
Hall symbol: P 2ac 2ab	Cell parameters from 3930 reflections
*a* = 7.533 (2) Å	θ = 2.9–26.4º
*b* = 7.701 (2) Å	µ = 0.13 mm^−^^1^
*c* = 19.288 (5) Å	*T* = 294 (2) K
*V* = 1118.9 (5) Å^3^	Block, colourless
*Z* = 4	0.24 × 0.22 × 0.18 mm

### Data collection

**Table:**

Bruker SMART CCD diffractometer	1359 independent reflections
Radiation source: fine-focus sealed tube	1280 reflections with *I* > 2σ(*I*)
Monochromator: graphite	*R*_int_ = 0.024
*T* = 294(2) K	θ_max_ = 26.4º
ω scans	θ_min_ = 2.1º
Absorption correction: multi-scan(SADABS; Bruker, 1997)	*h* = −9→6
*T*_min_ = 0.969, *T*_max_ = 0.977	*k* = −9→9
6331 measured reflections	*l* = −17→24

### Refinement

**Table:**

Refinement on *F*^2^	Hydrogen site location: difmap (N-H and O-H) and geom (C-H)
Least-squares matrix: full	H atoms treated by a mixture of independent and constrained refinement
*R*[*F*^2^ > 2σ(*F*^2^)] = 0.028	*w* = 1/[σ^2^(*F*_o_^2^) + (0.0475*P*)^2^ + 0.1468*P*] where *P* = (*F*_o_^2^ + 2*F*_c_^2^)/3
*wR*(*F*^2^) = 0.074	(Δ/σ)_max_ < 0.001
*S* = 1.06	Δρ_max_ = 0.25 e Å^−^^3^
1359 reflections	Δρ_min_ = −0.17 e Å^−^^3^
174 parameters	Extinction correction: SHELXL, Fc^*^=kFc[1+0.001xFc^2^λ^3^/sin(2θ)]^-1/4^
Primary atom site location: structure-invariant direct methods	Extinction coefficient: 0.067 (5)
Secondary atom site location: difference Fourier map	

### Special details

**Table:**

Geometry. All e.s.d.'s (except the e.s.d. in the dihedral angle between two l.s. planes) are estimated using the full covariance matrix. The cell e.s.d.'s are taken into account individually in the estimation of e.s.d.'s in distances, angles and torsion angles; correlations between e.s.d.'s in cell parameters are only used when they are defined by crystal symmetry. An approximate (isotropic) treatment of cell e.s.d.'s is used for estimating e.s.d.'s involving l.s. planes.
Refinement. Refinement of *F*^2^ against ALL reflections. The weighted *R*-factor *wR* and goodness of fit *S* are based on *F*^2^, conventional *R*-factors *R* are based on *F*, with *F* set to zero for negative *F*^2^. The threshold expression of *F*^2^ > σ(*F*^2^) is used only for calculating *R*-factors(gt) *etc*. and is not relevant to the choice of reflections for refinement. *R*-factors based on *F*^2^ are statistically about twice as large as those based on *F*, and *R*- factors based on ALL data will be even larger.

### Fractional atomic coordinates and isotropic or equivalent isotropic displacement parameters (Å^2^)

**Table:**

	*x*	*y*	*z*	*U*_iso_*/*U*_eq_	
O1	0.5541 (2)	0.9115 (2)	0.77366 (8)	0.0413 (4)	
H1	0.611 (4)	0.989 (4)	0.7999 (16)	0.062*	
O2	0.14604 (17)	0.39481 (16)	0.92596 (8)	0.0324 (3)	
O3	0.42454 (16)	0.30294 (16)	0.92082 (7)	0.0285 (3)	
O4	0.01406 (16)	0.07039 (17)	0.94283 (7)	0.0279 (3)	
H4	0.002 (3)	0.110 (3)	0.9859 (13)	0.042*	
O5	0.30427 (18)	0.05497 (16)	1.04183 (6)	0.0244 (3)	
H5	0.406 (3)	0.086 (3)	1.0507 (12)	0.037*	
O6	0.2651 (2)	−0.28770 (17)	0.91791 (7)	0.0324 (3)	
H6	0.218 (4)	−0.387 (4)	0.9212 (13)	0.049*	
O7	0.1836 (2)	−0.27117 (18)	1.02899 (7)	0.0380 (4)	
N1	0.6127 (2)	0.5901 (2)	0.84455 (8)	0.0280 (4)	
H1D	0.561 (3)	0.510 (3)	0.8145 (13)	0.042*	
H1E	0.665 (3)	0.529 (3)	0.8822 (13)	0.042*	
H1F	0.521 (3)	0.666 (3)	0.8599 (12)	0.042*	
C1	0.8957 (3)	0.5543 (4)	0.78287 (12)	0.0517 (6)	
H1A	0.8387	0.4739	0.7520	0.078*	
H1B	0.9459	0.4921	0.8213	0.078*	
H1C	0.9882	0.6146	0.7585	0.078*	
C2	0.7608 (3)	0.6838 (2)	0.80928 (9)	0.0288 (4)	
H2	0.8185	0.7610	0.8428	0.035*	
C3	0.6849 (3)	0.7920 (3)	0.75103 (10)	0.0333 (4)	
H3A	0.7805	0.8555	0.7289	0.040*	
H3B	0.6330	0.7154	0.7167	0.040*	
C4	0.2611 (2)	0.2769 (2)	0.92464 (8)	0.0198 (3)	
C5	0.1966 (2)	0.0871 (2)	0.92587 (8)	0.0204 (3)	
H5A	0.2126	0.0400	0.8791	0.025*	
C6	0.3130 (2)	−0.0200 (2)	0.97483 (8)	0.0202 (3)	
H6A	0.4360	−0.0185	0.9583	0.024*	
C7	0.2464 (2)	−0.2062 (2)	0.97795 (9)	0.0236 (4)	
O8	0.7125 (2)	0.1422 (2)	0.85702 (9)	0.0460 (4)	
H8A	0.633 (4)	0.190 (4)	0.8814 (15)	0.055*	
H8B	0.802 (4)	0.119 (4)	0.8783 (15)	0.055*	

### Atomic displacement parameters (Å^2^)

**Table:**

	*U*^11^	*U*^22^	*U*^33^	*U*^12^	*U*^13^	*U*^23^
O1	0.0473 (9)	0.0330 (7)	0.0436 (8)	0.0065 (7)	−0.0122 (7)	−0.0017 (7)
O2	0.0262 (6)	0.0181 (6)	0.0529 (8)	0.0018 (5)	0.0047 (6)	−0.0008 (6)
O3	0.0223 (6)	0.0244 (6)	0.0388 (7)	−0.0023 (5)	0.0013 (5)	0.0022 (6)
O4	0.0199 (6)	0.0271 (6)	0.0368 (7)	−0.0029 (6)	−0.0025 (5)	0.0002 (6)
O5	0.0243 (6)	0.0266 (6)	0.0224 (6)	−0.0045 (5)	−0.0013 (5)	−0.0019 (5)
O6	0.0449 (8)	0.0189 (6)	0.0333 (7)	−0.0063 (6)	0.0090 (6)	−0.0044 (5)
O7	0.0538 (9)	0.0273 (6)	0.0329 (7)	−0.0120 (7)	0.0100 (7)	0.0029 (6)
N1	0.0337 (9)	0.0260 (7)	0.0244 (7)	−0.0005 (8)	0.0006 (6)	0.0017 (6)
C1	0.0465 (13)	0.0641 (15)	0.0445 (12)	0.0226 (13)	0.0113 (10)	0.0148 (11)
C2	0.0276 (9)	0.0329 (9)	0.0259 (8)	−0.0018 (9)	−0.0010 (7)	0.0022 (7)
C3	0.0421 (11)	0.0314 (9)	0.0265 (8)	−0.0037 (9)	−0.0002 (8)	0.0062 (7)
C4	0.0244 (8)	0.0181 (7)	0.0169 (7)	−0.0002 (7)	−0.0004 (7)	0.0006 (6)
C5	0.0211 (7)	0.0177 (7)	0.0225 (7)	−0.0016 (6)	0.0008 (7)	−0.0008 (7)
C6	0.0195 (7)	0.0184 (7)	0.0228 (7)	−0.0003 (7)	0.0012 (6)	0.0000 (6)
C7	0.0230 (8)	0.0187 (8)	0.0291 (8)	0.0013 (7)	0.0000 (7)	0.0004 (7)
O8	0.0416 (9)	0.0528 (10)	0.0435 (9)	0.0115 (8)	−0.0096 (7)	−0.0171 (7)

### Geometric parameters (Å, °)

**Table:**

O1—C3	1.417 (3)	C1—C2	1.512 (3)
O1—H1	0.89 (3)	C1—H1A	0.9600
O2—C4	1.256 (2)	C1—H1B	0.9600
O3—C4	1.249 (2)	C1—H1C	0.9600
O4—C5	1.419 (2)	C2—C3	1.511 (3)
O4—H4	0.89 (2)	C2—H2	0.9800
O5—C6	1.4166 (19)	C3—H3A	0.9700
O5—H5	0.82 (3)	C3—H3B	0.9700
O6—C7	1.325 (2)	C4—C5	1.540 (2)
O6—H6	0.85 (3)	C5—C6	1.530 (2)
O7—C7	1.202 (2)	C5—H5A	0.9800
N1—C2	1.492 (2)	C6—C7	1.520 (2)
N1—H1D	0.93 (3)	C6—H6A	0.9800
N1—H1E	0.95 (3)	O8—H8A	0.85 (3)
N1—H1F	0.95 (3)	O8—H8B	0.81 (3)
			
C3—O1—H1	106 (2)	C2—C3—H3A	109.0
C5—O4—H4	106.3 (16)	O1—C3—H3B	109.0
C6—O5—H5	105.4 (17)	C2—C3—H3B	109.0
C7—O6—H6	108.7 (17)	H3A—C3—H3B	107.8
C2—N1—H1D	110.4 (16)	O3—C4—O2	124.44 (16)
C2—N1—H1E	106.4 (15)	O3—C4—C5	117.66 (15)
H1D—N1—H1E	108 (2)	O2—C4—C5	117.89 (14)
C2—N1—H1F	112.7 (15)	O4—C5—C6	111.34 (13)
H1D—N1—H1F	107 (2)	O4—C5—C4	113.31 (14)
H1E—N1—H1F	112 (2)	C6—C5—C4	109.88 (13)
C2—C1—H1A	109.5	O4—C5—H5A	107.3
C2—C1—H1B	109.5	C6—C5—H5A	107.3
H1A—C1—H1B	109.5	C4—C5—H5A	107.3
C2—C1—H1C	109.5	O5—C6—C7	109.44 (13)
H1A—C1—H1C	109.5	O5—C6—C5	108.48 (13)
H1B—C1—H1C	109.5	C7—C6—C5	110.12 (13)
N1—C2—C3	108.85 (16)	O5—C6—H6A	109.6
N1—C2—C1	109.68 (17)	C7—C6—H6A	109.6
C3—C2—C1	111.56 (16)	C5—C6—H6A	109.6
N1—C2—H2	108.9	O7—C7—O6	124.11 (15)
C3—C2—H2	108.9	O7—C7—C6	123.73 (15)
C1—C2—H2	108.9	O6—C7—C6	112.17 (14)
O1—C3—C2	113.08 (15)	H8A—O8—H8B	114 (3)
O1—C3—H3A	109.0		
			
N1—C2—C3—O1	57.1 (2)	C4—C5—C6—O5	58.54 (17)
C1—C2—C3—O1	178.27 (18)	O4—C5—C6—C7	51.92 (18)
O3—C4—C5—O4	169.20 (14)	C4—C5—C6—C7	178.28 (13)
O2—C4—C5—O4	−12.4 (2)	O5—C6—C7—O7	5.5 (2)
O3—C4—C5—C6	44.0 (2)	C5—C6—C7—O7	−113.67 (19)
O2—C4—C5—C6	−137.61 (15)	O5—C6—C7—O6	−174.79 (15)
O4—C5—C6—O5	−67.82 (17)	C5—C6—C7—O6	66.06 (18)

### Hydrogen-bond geometry (Å, °)

**Table:**

*D*—H···*A*	*D*—H	H···*A*	*D*···*A*	*D*—H···*A*
O1—H1···O8^i^	0.89 (3)	1.78 (3)	2.677 (2)	176 (3)
O4—H4···O3^ii^	0.89 (2)	2.01 (3)	2.885 (2)	169 (2)
O5—H5···O2^iii^	0.82 (3)	1.87 (3)	2.676 (2)	167 (3)
O6—H6···O2^iv^	0.85 (3)	1.77 (3)	2.6091 (19)	173 (3)
N1—H1D···O1^v^	0.93 (3)	2.05 (3)	2.945 (2)	159 (2)
N1—H1E···O5^iii^	0.95 (3)	1.91 (3)	2.852 (2)	168 (2)
N1—H1F···O6^i^	0.95 (3)	2.26 (3)	3.121 (2)	150 (2)
O8—H8A···O3	0.85 (3)	1.95 (3)	2.784 (2)	169 (3)
O8—H8B···O4^vi^	0.81 (3)	2.06 (3)	2.865 (2)	173 (3)

Symmetry codes: (i) *x*, *y*+1, *z*; (ii) *x*−1/2, −*y*+1/2, −*z*+2; (iii) *x*+1/2, −*y*+1/2, −*z*+2; (iv) *x*, *y*−1, *z*; (v) −*x*+1, *y*−1/2, −*z*+3/2; (vi) *x*+1, *y*, *z*.
